# APASL-ACLF without previous decompensation is immunologically distinct from EASL-CLIF-ACLF with previous decompensation

**DOI:** 10.3389/fimmu.2026.1893968

**Published:** 2026-10-06

**Authors:** Mona-May Langer, Marc J. Weiss, Alina Bauschen, Sabrina Guckenbiehl, Gerald Denk, Helena Stadler, Christian M. Lange

**Affiliations:** 1 Department of Internal Medicine II, LMU University Hospital, Munich, Germany; 2 Department for Gastroenterology and Hepatology, University Hospital Essen, University Duisburg-Essen, Essen, Germany; 3 Department of Internal Medicine I, University Medical Center of the Johannes Gutenberg University Mainz, Mainz, Germany

**Keywords:** acute-on-chronic liver failure, IL-17, liver cirrhosis, systemic inflammation, T cells

## Abstract

**Background and aims:**

Definitions of acute-on-chronic liver failure (ACLF) differ, with a focus on extrahepatic organ failures on the basis of previously compensated or decompensated cirrhosis in EASL-CLIF-ACLF versus hepatic failure in patients without previous decompensation in APASL-ACLF. Here, we explored whether patterns of systemic inflammation and immune cell alterations differ between these types of ACLF.

**Methods:**

Consecutive patients with acute decompensation, EASL-CLIF- or APASL-ACLF were recruited from two liver centres. Patterns of systemic inflammation were characterized by Olink analysis and changes in circulating immune cells by flow cytometry.

**Results:**

A total of 374 patients with acute decompensation (AD) were identified, of whom 56 fulfilled exclusively the EASL-CLIF-definition of ACLF (15% of all patients), while only 19 patients (5.1% of all patients) met exclusively the APASL-definition. APASL-ACLF was associated with higher bilirubin, lower creatinine, higher INR and higher hemoglobin concentrations compared to EASL-ACLF. Patterns of systemic inflammation differed between EASL-CLIF and APASL-ACLF. For example, FGF-23, IL-17 or VEGF-A were associated with EASL-CLIF-ACLF, while FGF-19 or low IL-12 were associated with APASL-ACLF. Moreover, patients with EASL-CLIF-ACLF had significantly lower counts of CD8+ T cells, while in APASL-ACLF a reduction of CD4+ T cell was characteristic. Detailed immune cell phenotyping revealed distinct expression of co-stimulatory molecules, inhibitory molecules and T cell activation markers in APASL-ACLF compared to patients with AD or EASL-CLIF-ACLF.

**Conclusion:**

We provide preliminary evidence that EASL-CLIF- and APASL-ACLF are associated with different changes in systemic inflammation patterns and immune cell phenotypes, suggesting that both types of ACLF might be pathophysiologically different.

## Introduction

Compensated liver cirrhosis is characterized by the absence of cirrhosis-associated complications like ascites, variceal bleeding or hepatic encephalopathy (1). New onset of these complications defines the transition from compensated to decompensated cirrhosis, which is associated with a significant increase in the risk of liver-related mortality. Decompensation events of cirrhosis can occur as either chronic or acute decompensation (AD) (2). Extrahepatic organ dysfunction, which is typically triggered by further deterioration of liver function, bleeding or infection, defines acute-on-chronic liver failure (ACLF), that is associated with an additional increase in the risk of short-term mortality (3).

Yet, international definitions of ACLF differ. While the EASL-CLIF definition of ACLF is based on the presence AD in combination with specific, mainly extrahepatic organ failures (3), the APASL definition focusses on the hepatic reserve and defines ACLF as an acute hepatic insult manifesting as jaundice and coagulopathy, complicated by new onset of ascites and/or encephalopathy in patients with pre-existing chronic liver disease (4). Another very important difference between EASL-CLIF and APASL-ACLF is, that prior decompensation does not preclude defining EASL-CLIF-ACLF, while per definition APASL-ACLF can only be diagnosed in patients with previously compensated liver cirrhosis (3, 4). Moreover, there are methodologic differences between both concepts of ACLF. While the development of the EASL-CLIF-ACLF concept has been outcome-based, applying thresholds of a high short-term-mortality, the APASL-ACLF definition was established on a consensus-based system. Lastly, NASCELD definition of ACLF shares similarities with the EASL definition, as it is defined by acute deterioration of cirrhosis in terms of developing two or more extrahepatic organ failures on the basis of previously compensated or decompensated cirrhosis (5).

Definitions of ACLF have been derived from clinical considerations, in particular by recognizing that the syndrome of ACLF is associated with high short-term mortality. Notably, extensive research has now revealed important pathogenetic processes which contribute to the development of ACLF and it´s mortality, namely systemic inflammation and mitochondrial dysfunction (2, 6–9). In the present study, we aimed to explore whether patterns of systemic inflammation and immune cell alterations differ between patients who fulfil the definition of EASL-CLIF or APASL-ACLF in order to understand whether the focus on liver versus extrahepatic organ failures and the presence of prior decompensation versus ACLF as first manifestation of decompensation might reflect pathophysiologically distinct patient groups.

## Patients and methods

### Patients

For the present study, hospitalized patients with liver cirrhosis who were admitted to the University Hospital Essen, Germany, or to the LMU University Hospital Munich, Germany, from October 2018 to May 2023, were included. Written informed consent was obtained from all participants, and ethical approval was obtained from the local ethical committees (WBE-071 and LMU 22-0392). Pregnant and breast-feeding patients, as well as patients with hepatocellular carcinoma beyond Milan criteria or patients tested positive for human immunodeficiency virus were excluded from the study. AD or ACLF were classified according to the criteria of the EASL-CLIF consortium and according to the APASL-criteria (2) (3, 4),. Blood samples for all analysis were collected at baseline of hospitalization, when classification of patients was performed.

### Analysis of inflammatory plasma proteins and mediators by proximity extension analysis assay

Quantification of 92 human plasma proteins by proximity extension analysis assay (Olink Target 96 Inflammation Panel, Olink, Uppsala, Sweden) was performed as described previously (10). In total, 76 of 92 proteins passed quality control, were above the limit of detection and could be included in the present analysis. Of note, we have published Olink data in a previous analysis of thyroid hormones in liver cirrhosis (10) from 70 out of 138 patients included in the present analysis.

### Flow cytometry of immune cells

Immunophenotyping was performed with peripheral blood mononuclear cells (PBMCs) of 6 patients with acute decompensation, 6 patients with EASL-CLIF-ACLF and 7 patients with APASL-ACLF. PBMCs were isolated using density gradient centrifugation separation (Pancoll human separation solution, PAN-Biotech). For flow cytometry, cells were incubated with an FcR-Blocking reagent (Miltenyi Biotec) and then stained with fluorochrome-coupled antibodies in FACS-staining buffer (PBS + 0.5% BSA). Cellular viability was estimated using the Zombie UV Viability Antibody (BioLegend). Quantification of subpopulation frequencies of the innate and adaptive immune system was done according to recommendations for immunophenotyping in flow cytometry by the Human Immunology Project consortium (11). For T-lymphocytes, several activation and inhibitory markers were looked at after gating for CD4+ and CD8+ T-cells. Fluorescence-minus-one (FMO-)-controls were done beforehand to correctly determine cellular expression levels. The gating strategy for T-lymphocytes is shown in **Supplementary Information Figure 1**.

### Statistical analysis

Statistical analysis was performed using GraphPad Prism v9.3.1 software (GraphPad Software, San Diego, CA, USA). After testing for Gaussian distribution, two groups were compared by T-test or Wilcoxon-Mann-Whitney-U-Test, as appropriate, while comparison of more groups was done by One-way ANOVA or Kruskal-Wallis-Test, as appropriate. *P* values less than 0.05 were considered to be statistically significant. Benjamini-Hochberg FDR correction was applied to correct for multiple testing. Graphs were built using GraphPad Prism v9.3.1 software and Data was further analyzed using Python (version 3.13.2) with seaborn and matplotlib library as well as RStudio with ComplexUpset package.

## Results

### Patient characteristics

A total of 374 patients with AD were identified, of whom 130 fulfilled the definition of any ACLF (EASL-CLIF or APASL). Of those, 56 met exclusively the EASL-CLIF but not the APASL-definition of ACLF (15% of all patients with AD), while only 19 patients (5.1% of all patients with AD) fulfilled exclusively the full APASL-definition and not the EASL-CLIF definition. An additional 11 patients would meet the APASL-definition (but not the EASL-CLIF definition) of ACLF, if the criterion “previously compensated cirrhosis” would be neglected (details in **Figure 1**). 43 patients fulfilled the definitions of both EASL-CLIF- and APASL-ACLF. Patients with APASL-ACLF had higher bilirubin concentrations, lower creatinine concentrations, higher INR and higher hemoglobin concentrations compared to patients with EASL-ACLF. Detailed baseline characteristics of all patients are shown in **Table 1**, and baseline characteristics of patients with APASL-ACLF without versus with previous decompensation are shown in **Supplementary Information Table 1**, while baseline characteristics of patients with mixed ACLF are shown in **Supplementary Information Table 2**.

**Figure 1:**
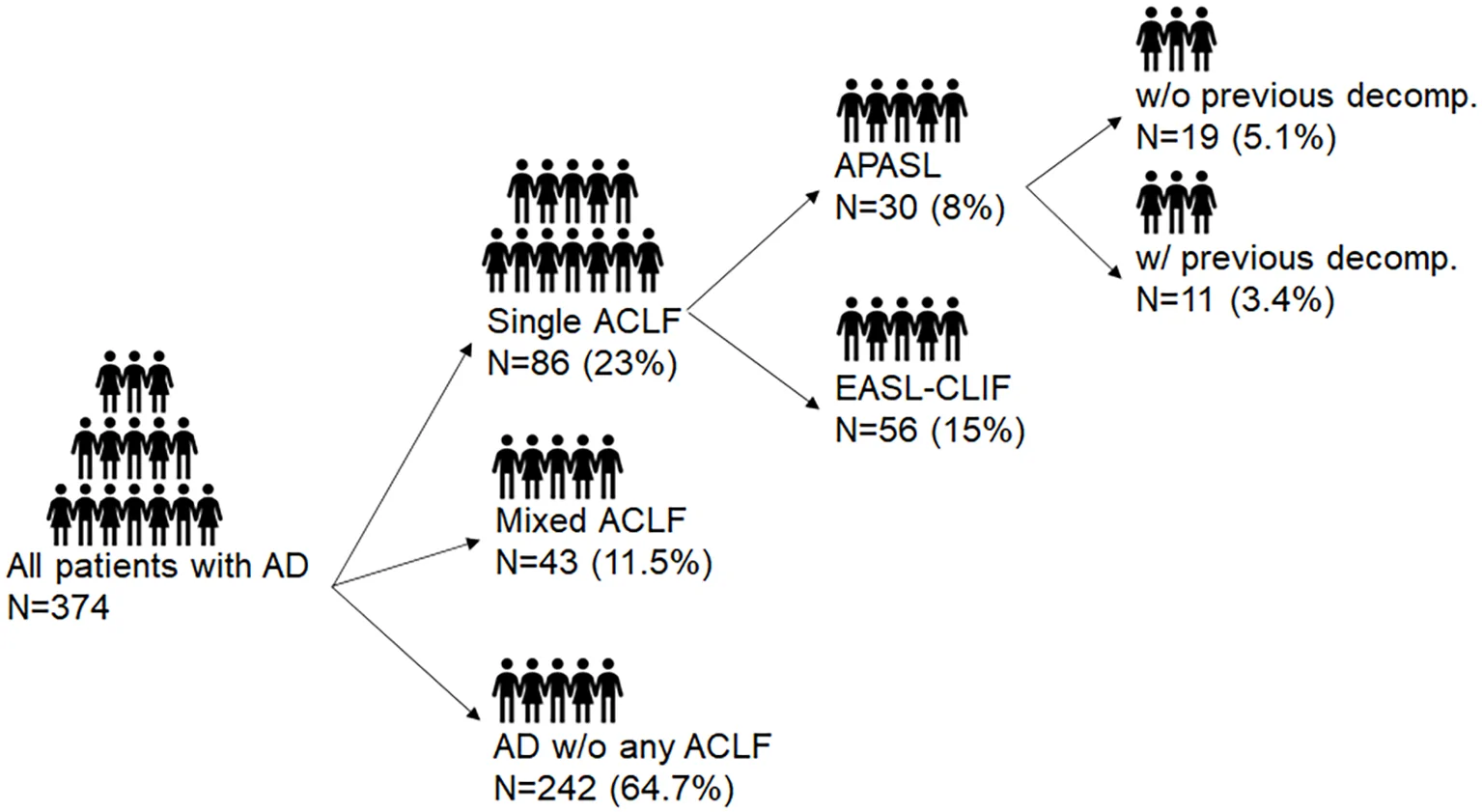
Distribution of acute-on-chronic liver failure (ACLF)-types (EASL versus APASL) among hospitalized patients with acute decompensation (AD). Single ACLF refers to patients who exclusively fulfill either the EASL-CLIF or the APASL-definition of ACLF, whereas mixed ACLF refers to patients who fulfill both definitions of ACLF. Percentages indicate the proportion relative to all patients with AD.

**Table 1:** Baseline characteristics.

Variable	AD (N = 242)	APASL w/o prev. decomp (N = 19)	EASL-CLIF exclusively ACLF (N = 56)*	P-value AD vs APASL	P-value AD vs EASL-CLIF	P-value APASL vs EASL-CLIF
Age [years], mean (SD)	56 (0.7)	52 (2.4)	57 (1.3)	0.2	0.7	0.1
Female gender; male gender, N (%)	87 (36); 155 (64)	8 (42); 11 (58)	15 (27); 41 (73)	0.6	0.2	0.2
Child Pugh Score, mean (SD)	8.2 (0.1)	10.1 (0.4)	8.7 (0.2)	**0.0001**	0.1105	**0.03**
CLIF OF score, mean (SD)	7.8 (0.1)	9.5 (0.4)	10.2 (0.3)	**0.0001**	**<0.0001**	>0.9999
MELD score, mean (SD)	15.0 (0.3)	23.7 (0.7)	24.1 (1.0)	**<0.0001**	**<0.0001**	>0.9999
Etiology of liver cirrhosis						
Viral, N (%)	25 (10.3)	0 (0.0)	5 (8.9)	0.1	0.7	0.2
NASH, N (%)	26 (10.7)	0 (0.0)	8 (14.3)	0.1	0.5	0.08
Alcoholic, N (%)	108 (44.6)	12 (63.2)	34 (60.7)	0.1	**0.03**	0.9
Cholestatic, N (%)	25 (10.3)	2 (10.5)	2 (3.6)	0.98	0.1	0.2
Others, N (%)	48 (19.8)	4 (21.1)	5 (8.9)	0.9	0.05	0.2
Mixed etiologies, N (%)	10 (4.1)	1 (5.3)	2 (3.6)	0.8	0.9	0.7
Clinical biochemistry						
Leukocytes [per nL], mean (SD)	6.7 (0.3)	9.7 (1.5)	8.6 (0.8)	0.08	0.07	>0.9999
Hemoglobin [g/dL], mean (SD)	12.6 (1.0)	10.1 (0.5)	8.7 (0.2)	>0.9999	**<0.0001**	0.08
Platelets [per nL], mean (SD)	131.5 (7.2)	109.7 (14.9)	113.6 (10.6)	>0.9999	0.9	>0.9999
CRP [mg/dL], mean (SD)	3.2 (0.4)	2.7 (0.6)	4.8 (0.6)	>0.9999	**0.0007**	0.3
Sodium [mmol/L], mean (SD)	136.0 (0.3)	133.6 (1.2)	134.8 (0.8)	0.2	0.4	>0.9999
Creatinine [mg/dL], mean (SD)	1.1 (0.02)	1.0 (0.07)	2.7 (0.2)	>0.9999	**<0.0001**	**<0.0001**
Bilirubin [mg/dL], mean (SD)	3.8 (0.3)	14.7 (2.3)	8.2 (1.5)	**<0.0001**	>0.9999	**<0.0001**
AST [U/L], mean (SD)	76.2 (6.0)	108.9 (24.0)	67.0 (7.4)	0.09	>0.9999	0.08
GGT [U/L], mean (SD)	191.4 (25.6)	145.9 (41.8)	160.8 (24.4)	>0.9999	>0.9999	>0.9999
INR, mean (SD)	1.4 (0.02)	1.8 (0.1)	1.5 (0.06)	**<0.0001**	**0.0317**	**0.0008**
Albumin [g/dL], mean (SD)	3.2 (0.1)	3.1 (0.2)	3.1 (0.1)	0.9	0.9	0.9
*Hepatic encephalopathy*						
Grade 0, N (%)	200 (82.6)	17 (89.5)	38 (67.9)	0.4	**0.01**	0.06
Grade 1, N (%)	23 (9.5)	1 (5.3)	6 (10.7)	0.5	0.8	0.5
Grade 2, N (%)	13 (5.4)	1 (5.3)	9 (16.1)	0.98	**0.006**	0.2
Grade 3, N (%)	6 (2.5)	0 (0.0)	3 (5.4)	0.5	0.3	0.3
***Gastrointestinal bleeding*,** N (%)	25 (10.3)	1 (5.3)	8 (14.3)	0.5	0.4	0.3
***Infections***, N (%)	72 (29.8)	12 (63.2)	35 (62.5)	0.003	**<0.0001**	0.96
*Ascites*						
No ascites, N (%)	84 (34.7)	0 (0.0)	12 (21.4)	**0.002**	0.06	**0.03**
Moderate, N (%)	40 (16.5)	7 (36.8)	9 (16.1)	**0.03**	0.9	0.06
Massive, N (%)	118 (48.8)	12 (63.2)	35 (62.5)	0.2	0.06	0.96
Outcome						
Mortality within 28 d, N (%)	12 (5.0)	1 (5.3)	6 (10.7)	0.95	0.1	0.5

P-values were calculated with One-way ANOVA, Kruskal-Wallis or Chi^2^ -Test, as appropriate.

AP, alkaline phosphatase; AST, aspartate aminotransferase; CLIF-OF, chronic liver failure – organ failure; CRP, C-reactive protein; GGT, y-glutamyl transferase; Hb, hemoglobin; INR, international normalized ratio; MELD, model of end-stage liver disease.

*57%, 37%, and 6% of patients with EASL-CLIF-ACLF had ACLF grade 1, 2, and 3, respectively. Significant P values are in bold.

### Patterns of systemic inflammation differ between EASL-CLIF- and APASL-ACLF

To explore whether patients with EASL-CLIF versus APASL-ACLF exhibit different patterns of systemic inflammation, plasma proteins and inflammatory mediators were quantified in baseline samples of 138 patients by proximity extension analysis assay. Baseline characteristics of this subgroup are shown in **Supplementary Information Table 3**, including patients with mixed type ACLF, and are representative for the whole cohort. In line with previous observations, higher levels of a number of mediators were observed in patients who exclusively fulfilled the EASL-CLIF-ACLF (but not APASL) criteria compared to patients with AD (**Figure 2**). Patients who exclusively fulfilled the APASL-ACLF (but not EASL-CLIF) criteria also showed increased levels of inflammatory mediators compared to patients with AD without any ACLF, but patterns of systemic inflammation appeared to be different between EASL-CLIF and APASL-ACLF (**Figures 2**, **3**). Examples of mediators of potential functional interest which were predominantly associated with EASL-CLIF-ACLF included FGF-23, IL-17 or VEGF-A, while for example FGF19 or downregulation of IL-12 were associated with APASL-ACLF. Yet, statistical significance was lost after correction for multiple testing, probably due to the low number of patients with APASL-ACLF (details in **Figures 2**, **3**). In line, **Supplementary Information Figure 2** shows that increased creatinine, typical for EASL-CLIF-ACLF, correlates with other soluble mediators than increased bilirubin (predominant in APASL-ACLF).

**Figure 2:**
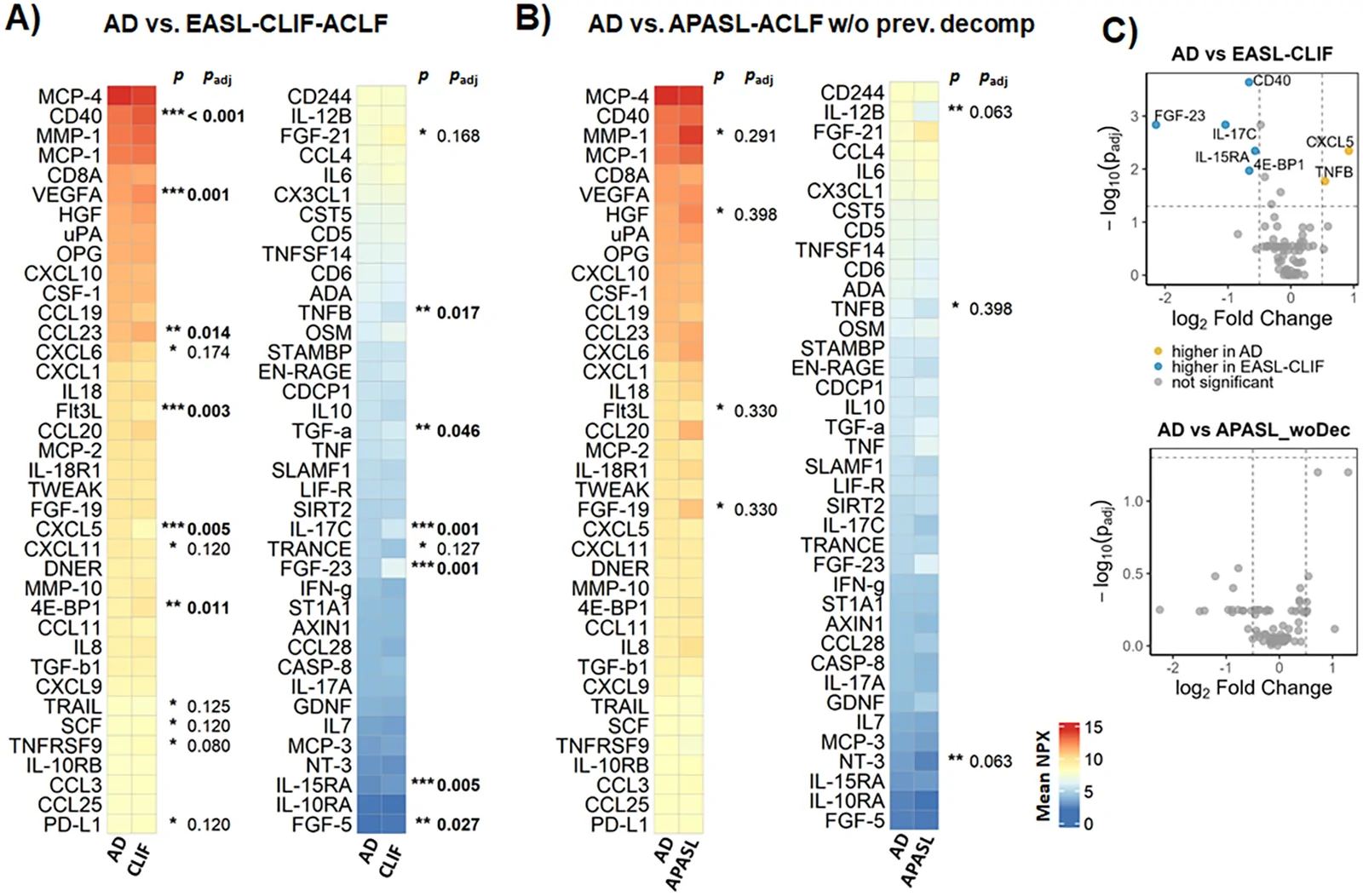
Patterns of systemic inflammation according to the type of ACLF. Heat-maps of 76 plasma proteins and inflammatory mediators which were quantified by proximity extension analysis assay (Olink), in patients with AD versus EASL-CLIF-ACLF **(A)** and in patients with AD versus APASL-ACLF **(B)**. **(C)** Volcano blots of plasma proteins and mediators differentially regulated (dots in colour mark differentially regulated, i.e. statistically different mediators) according to type of AD / ACLF. Unadjusted *P ≤ 0.05, **P ≤ 0.01, ***P ≤ 0.001. Adjusted P-values are shown in the right column.

**Figure 3:**
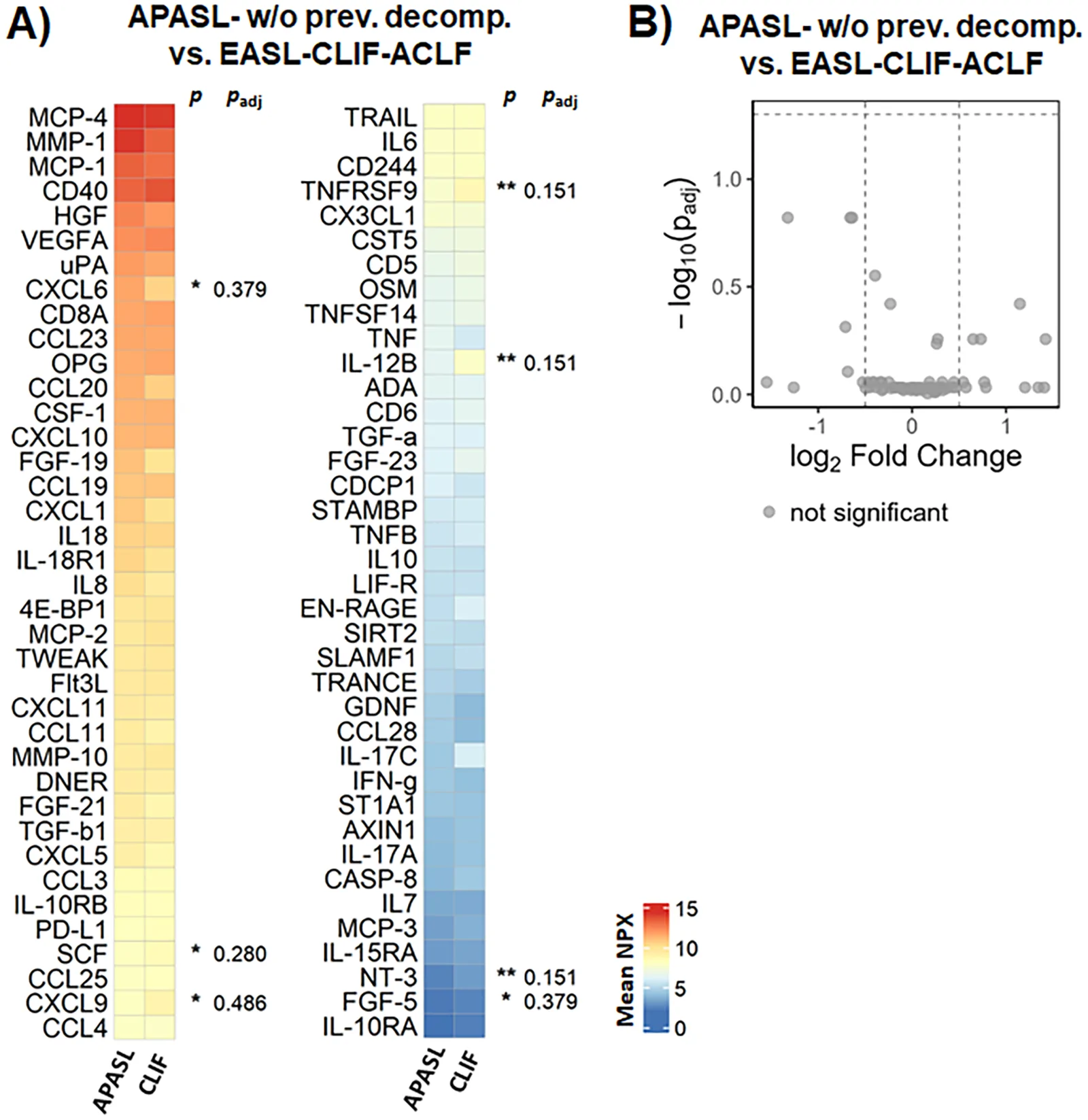
Patterns of systemic inflammation in patients with EASL-CLIF-ACLF vs. APASL-ACLF. Heat-maps of 76 plasma proteins and inflammatory mediators which were quantified by proximity extension analysis assay (Olink) in patients with APASL- versus EASL-ACLF **(A)**. **(B)** Volcano blots of plasma proteins and mediators. Unadjusted *P ≤ 0.05, **P ≤ 0.01. Adjusted P-values are shown in the right column.

Since the exclusion of prior decompensation is unique for the APASL definition of ACLF, we assessed whether patterns of systemic inflammation differ in patients with APASL-ACLF without prior decompensation compared to patients who would meet the APASL-criteria of liver failure but on a background of previous decompensation. As shown in **Supplementary Information Figure 3**, patterns of systemic inflammation might be partially different between both groups, suggesting that previous decompensation affects patterns of systemic inflammation during the development of ACLF. Finally, we assessed whether patterns of systemic inflammation are different between patients with EASL-CLIF-ACLF vs. mixed-ACLF (i.e. patients who fulfilled both definitions of ACLF). Of note, systemic inflammation appeared to be highest in patients with mixed-ACLF (**Figure 4**).

**Figure 4:**
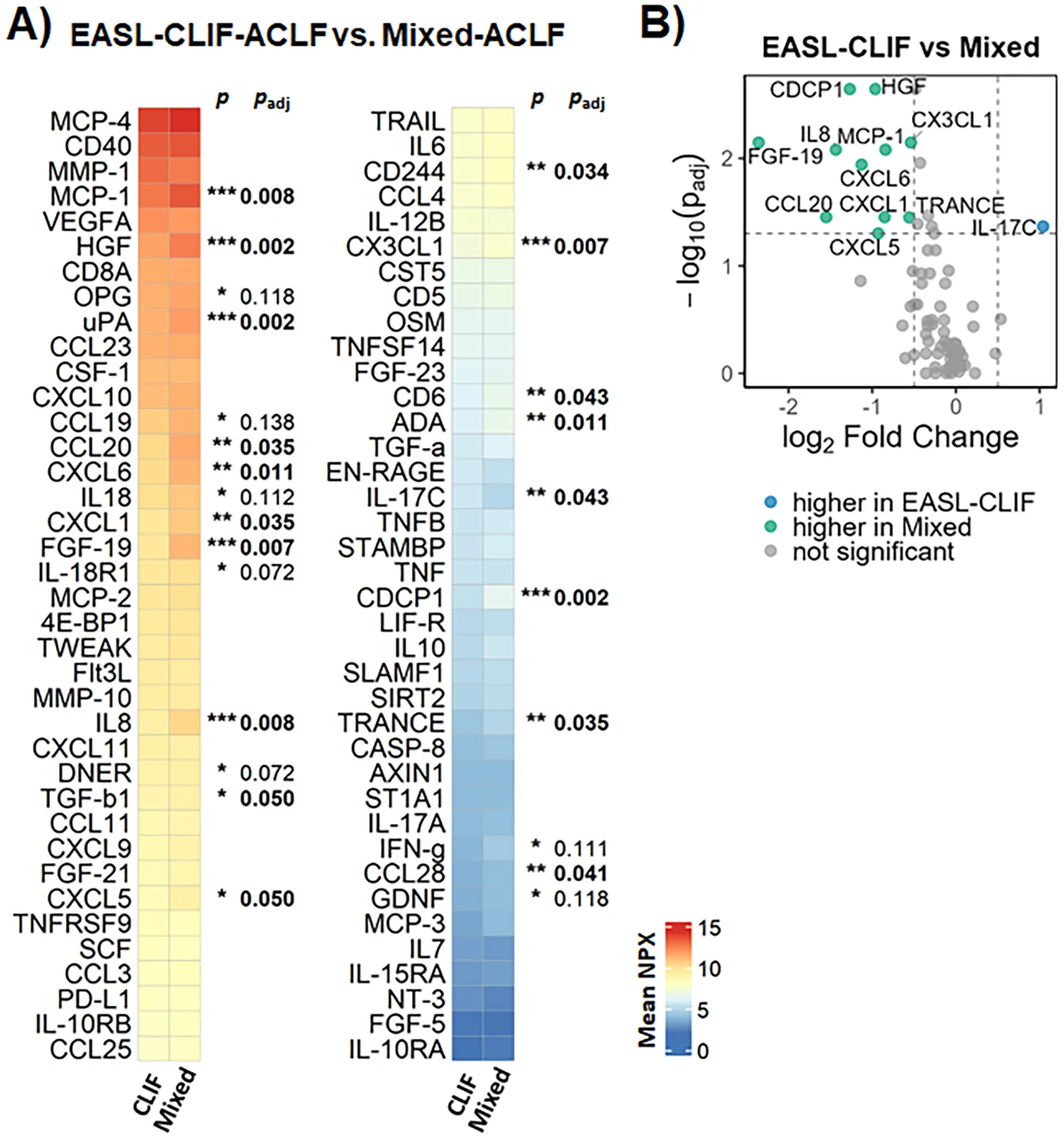
Patterns of systemic inflammation in patients with EASL-CLIF-ACLF vs. mixed-ACLF. **(A)** Heat-maps of 76 plasma proteins and inflammatory mediators which were quantified by proximity extension analysis assay (Olink) in patients with EASL-CLIF-ACLF versus mixed-ACLF (i.e. patients who fulfilled the definitions of both APASL- and EASL-ACLF. **(B)** Volcano blots of plasma proteins and mediators. Unadjusted *P ≤ 0.05, **P ≤ 0.01, ***P ≤ 0.001. Adjusted P-values are shown in the right column.

### EASL-CLIF- versus APASL-ACLF are associated with distinct defects in peripheral immune cells

We next quantified key populations of the innate and adaptive immune system in blood of a subgroup of patients (N = 13) with available PBMCs fulfilling different definitions of ACLF. Baseline characteristics of this subgroup are shown in **Supplementary Information Table 4** and are representative for the whole cohort. Six additional patients with acute decompensation and neither EASL-CLIF- nor APASL-ACLF were used as a reference. No relevant differences in frequencies of B cells as well as in frequencies of NK cells or monocyte populations were observed between patients with EASL-CLIF-ACLF versus APASL-ACLF (**Supplementary Information Figure 4**). In contrast, patients with EASL-CLIF-ACLF had significantly lower counts of CD8+ T cells compared to patients with APASL-ACLF, while in patients with APASL-ACLF, CD4+ T cell counts were significantly lower compared to EASL-CLIF-ACLF (**Figure 5**). Of note, changes in the composition of CD4+ and CD8+ T cells in patients with AD without any ACLF were largely comparable to that of patients with EASL-CLIF-ACLF, but fundamentally different from patients with APASL-ACLF (**Figure 5**). Differences in subpopulations of CD4+ T cells, like central memory T cells or effector T cells, were rather minor between different patient groups, with the notable exception of higher Th17-cells in AD/EASL-CLIF-ACLF versus APASL-ACLF (**Supplementary Information Figure 5**). CD8+ T-cell subpopulations showed distinct differences between the groups, with naïve T-cells becoming the most dominant subset in APASL-ACLF, whilst effector- and effector-memory T-cells diminished. Detailed phenotyping of the expression of cell surface markers revealed clearly distinct expression of co-stimulatory molecules, inhibitory molecules and T cell activation markers on CD4+ T cells and CD8+ T cells of patients with APASL-ACLF compared to patients with AD without ACLF and CLIF-ACLF (**Figure 6**).

**Figure 5:**
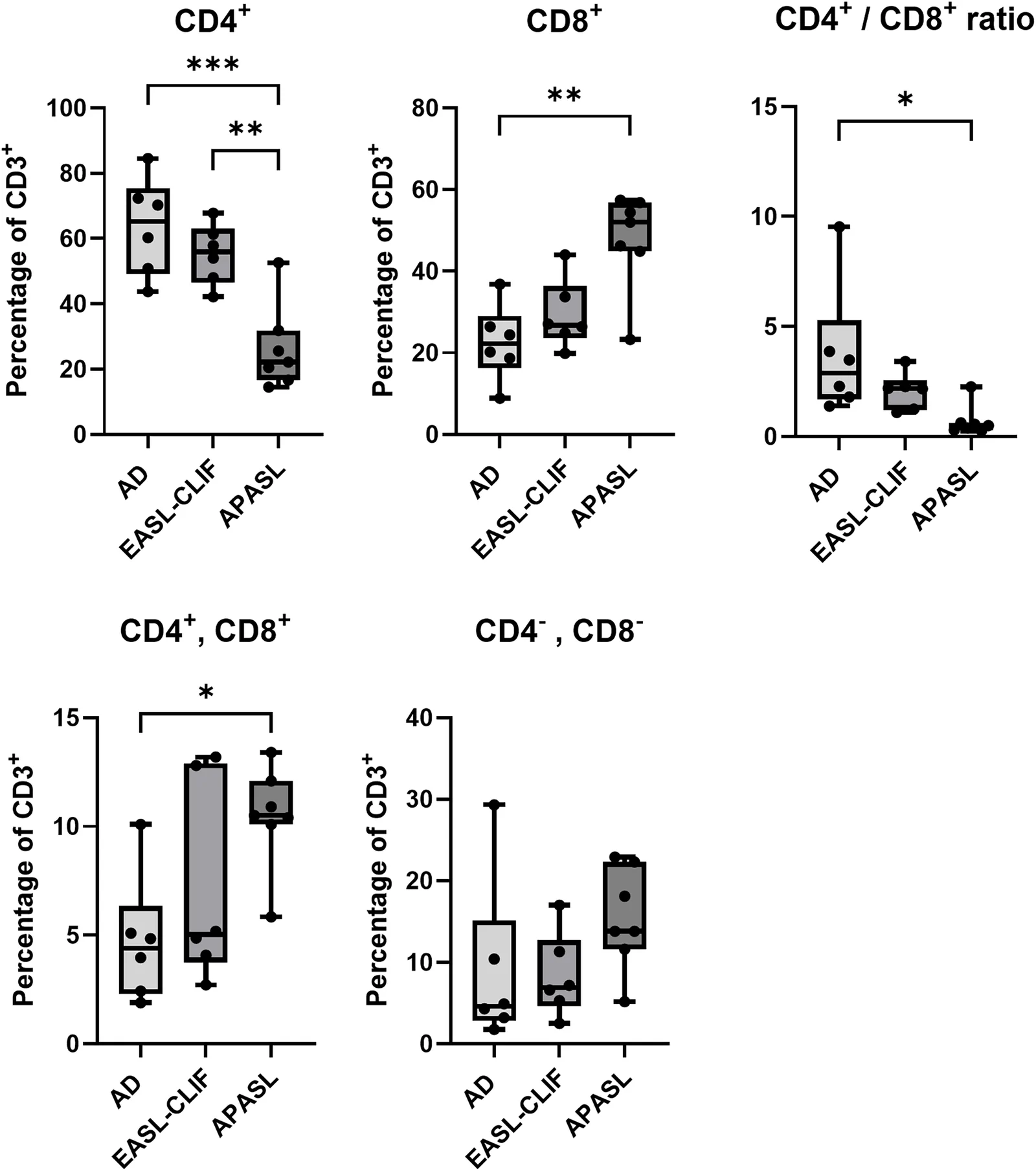
Frequencies of T cell populations according to the type of ACLF. All frequencies are presented as frequency of CD3+ T-lymphocytes. T-lymphocytes were subdivided into 4 subsets according to CD4 and CD8 co-expression. *P<=0.05; **P<=0.01; ***P<=0.001.

**Figure 6:**
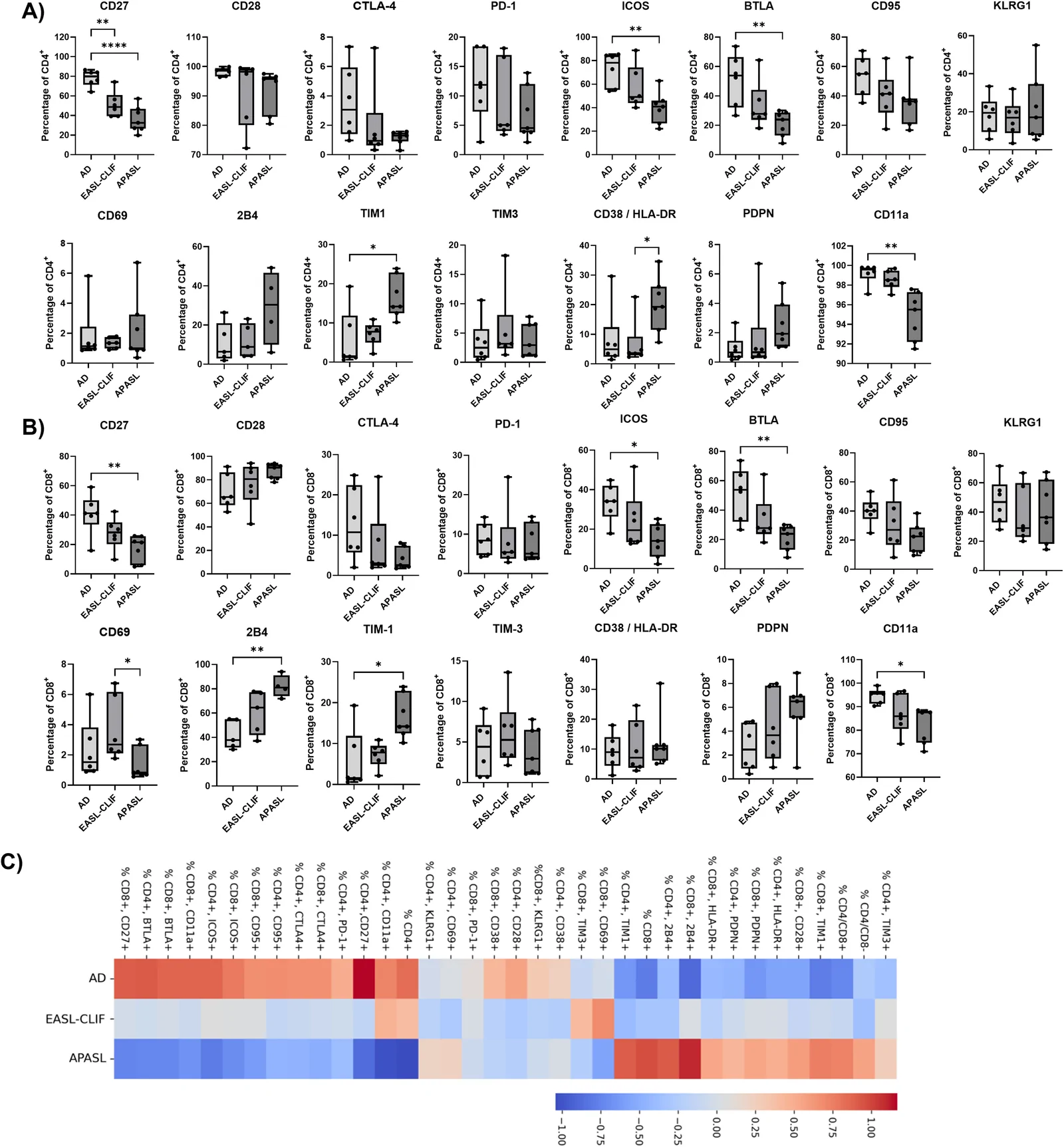
Expression of cell surface markers on CD4+ T cells and CD8+ T cells. **(A)** Expression levels on CD4+ T-cells of activation markers (CD27, CD28, CD38/HLA-DR, CD95, CD69, ICOS) and inhibitory markers (CTLA-4, PD-1, BTLA, TIM-1, TIM-3, KLRG1, 2B4), as well as adhesion proteins (CD11a) in patients with acute decompensation (AD), EASL-CLIF-ACLF and APASL-ACLF. **(B)** Expression levels of these markers on CD8+ T-cells in patients with AD, EASL-CLIF-ACLF or APASL-ACLF. **(C)** Z-normalized heatmap of marker expressions according to the relative expression of the 3 groups. *P<=0.5, **P<=0.01, ****P<=0.0001.

## Discussion

Although current attempts aim to unify international definitions of ACLF, there are significant conceptual differences between classical definitions of EASL-CLIF- and APASL-ACLF, namely with respect to the exclusion of previous decompensation in APASL-ACLF and with respect to the focus on extrahepatic versus hepatic organ failures in EASL-CLIF- versus APASL-ACLF. It was assumed by some authors, that the different definitions of ACLF apply for patients at different stages of the same syndrome. In this regard, the APASL-definition would identify patients at an earlier stage of the syndrome than the EASL-CLIF-definition (5). Yet, it is also plausible that differences between the definitions of ACLF are partially driven by a different spectrum of underlying causes of ACLF in Western versus Asian countries, like a much higher frequency of Hepatitis B-related ACLF in Asia compared to the Western world (12). Of note, a recent study has shown that expression of components of the lipopolysaccharide (LPS) response pathway such as CD180, LY96, or VCAM-1 are aetiology-dependent in liver cirrhosis, with substantial differences between metabolic, autoimmune, or viral aetiology (13). Of note, clinical concepts of ACLF are currently under revision and novel, uniform definitions are on the horizon (14, 15). In this regard, it is noteworthy that in our study in Western patients, only a small proportion of patients with any ACLF fulfilled the APASL- but not the EASL-definition of ACLF. Clinical features, which were associated with APASL-ACLF in our cohort were consistent with expected higher concentrations of bilirubin and INR as well as with much lower concentrations of creatinine. These differences might explain differences in standard clinical scores in both groups: While in APASL-ACLF, the mean Child Pugh Score was higher compared to EASL-CLIF-ACLF, mean MELD-score was higher in EASL-CLIF-ACLF compared to APASL-ACLF. Moreover, hemoglobin concentration was significantly lower in EASL-CLIF- versus APASL-ACLF. Of note, patients who fulfilled both definitions of ACLF (i.e. mixed ACLF) had the highest mortality and the highest proportion of ACLF grade 3 in our cohort, in line with highest levels of systemic inflammation.

Beyond these clinical considerations, we provide preliminary evidence that patterns of systemic inflammation as well as changes in key immune cell populations differ between EASL-CLIF- and APASL-ACLF. A contribution of systemic inflammation to the pathogenesis of ACLF has been recognized in studies addressing both EASL-CLIF- and APASL-ACLF (9, 16). Systemic inflammation is considered to promote organ dysfunction in ACLF, partially by inducing mitochondrial dysfunction. Furthermore, prolonged states of systemic inflammation can induce exhaustion and dysfunction of cellular immune responses (17). Indeed, impaired innate immune cell responses, which can occur rapidly in states of systemic inflammation, have been described in patients with both EASL-CLIF or APASL-ACLF (18–20). In line, we have not observed relevant differences in key innate immune cell populations between patients with EASL-CLIF- and APASL-ACLF. In contrast, we have observed fundamental differences in major populations of the adaptive immune system. Most prominently, patients with AD or with EASL-CLIF-ACLF showed lower frequencies of CD8+ T cells compared to patients with APASL-ACLF, while frequencies of CD4+ T cells showed an inverse behaviour. A reduction of CD8+ T cells and an expansion of certain CD4+ T cell populations like HLA-G-positive suppressor CD4+ T cells has been described previously in patients with AD without ACLF, suggesting that decompensated cirrhosis results in profound alterations of the immune system which precede EASL-ACLF and may contribute to different and more severe exhaustion of cellular immune responses in EASL-ACLF compared to APASL-ACLF (21–23). In contrast, upregulation of B- and T-lymphocyte attenuator (BTLA) on CD4+ T cells has been identified to contribute to CD4+ T cell loss and exhaustion in patients with APASL-ACLF (24). A recognition of differences in immune function between EASL-CLIF- and APASL-ACLF, and of the contribution of prior decompensation to such differences, might help to design appropriate treatment modalities for different forms or stages of ACLF.

In addition to differences in the adaptive immune system, we have observed potentially interesting differences in some important soluble mediators between EASL-CLIF and APASL-ACLF. For example, APASL-ACLF was associated with much lower IL-12B concentrations than EASL-CLIF-ACLF. IL-12B is an important cytokine, contributing for example to induction of Th1 cells and maintenance of effector memory T cells. Indeed, an accumulation of effector memory T cells has been observed in EASL-CLIF-ACLF and may contribute to uncontrolled systemic inflammatory responses (23). Additional, remarkable differences were observed within members of the FGF family. While EASL-CLIF-ACLF was associated with high FGF-23 concentrations, APASL-ACLF was associated with increased FGF19 concentrations. These observations appear plausible from a biologic point of view, as FGF23 is strongly related to kidney failure and its sequalae, while FGF19 is associated with bile acid metabolism (25, 26). Finally, EASL-CLIF-ACLF was associated with both an increase of Th17 cells in blood and with increased IL-17 serum concentrations. Yet, these findings have to be confirmed in larger studies, since the statistical significance was lost after correction for multiple testing, probably due to the low sample size of patients with APASL-ACLF.

Our study has several limitations. Most importantly, the sample size of patients with APASL-ACLF is relatively small, due to the rare presentation of this entity in a large European reference centre. In particular, the low number of patient samples which could be included in the flow cytometry analyses of cellular immune phenotypes is small and further studies are needed to fully confirm our findings.

Collectively, we show that EASL-CLIF- and APASL-ACLF are associated with different changes in systemic inflammation patterns and changes in circulating immune cell populations. These changes appear to be partially driven by prior decompensation in patients with EASL-ACLF- compared to patients with APASL-ACLF, suggesting that this criterion is important for the understanding of clinical and pathophysiological differences between both definitions.

## Data Availability

The original contributions presented in the study are included in the article/**Supplementary Material**. Further inquiries can be directed to the corresponding author.
