# Supplementary material for: APASL-ACLF without previous decompensation is immunologically distinct from EASL-CLIF-ACLF with previous decompensation
Source: Front Immunol. 2026 Oct 6;17:1893968. doi: 10.3389/fimmu.2026.1893968 (PMC13680246; doi:10.3389/fimmu.2026.1893968)
Supplement: Supplementary file 1 [file DataSheet1.pdf]

SI Information

SI Table 1. Baseline concentrations of patients with "APASL-ACLF" with versus without previous decompensation

	APASL w/o prev. decomp (N=19)	APASL w/ prev. decomp (N=11)	P-value
Age [years], mean (SD)	52 (2.4)	58 (2.4)	0.08
Female gender; male gender, N (%)	8 (42); 11 (58)	3 (27); 8 (73)	0.4
Child Pugh Score, mean (SD)	10.1 (0.4)	9.9 (0.3)	0.7
CLIF OF score, mean (SD)	9.5 (0.4)	9.4 (0.6)	0.7
MELD score, mean (SD)	23.7 (0.7)	24.2 (0.9)	0.6
**Etiology of liver cirrhosis**			
Viral, N (%)	0 (0.0)	0 (0.0)	-
MASH, N (%)	0 (0.0)	0 (0.0)	-
Alcoholic, N (%)	12 (63.2)	9 (81.8)	0.3
Cholestatic, N (%)	2 (10.5)	0 (0.0)	0.3
Others, N (%)	4 (21.1)	1 (9.1)	0.4
Mixed etiologies, N (%)	1 (5.3)	1 (9.1)	0.7
**Clinical biochemistry**			
Leukocytes [per nL], mean (SD)	9.7 (1.5)	6.4 (1.1)	0.2
Hemoglobin [g/dL], mean (SD)	10.1 (0.5)	9.3 (0.8)	0.4
Platelets [per nL], mean (SD)	109.7 (14.9)	78.4 (13.0)	0.2
CRP [mg/dL], mean (SD)	2.7 (0.6)	1.8 (0.6)	0.2
Creatinine [mg/dL], mean (SD)	1.0 (0.07)	1.0 (0.1)	0.9
Bilirubin [mg/dL], mean (SD)	14.7 (2.3)	11.3 (1.9)	0.6
AST [U/L], mean (SD)	108.9 (24.0)	76.8 (11.0)	0.5
GGT [U/L], mean (SD)	145.9 (41.8)	93.73 (30.5)	0.5
AP [U/L], mean (SD)	148.4 (14.7)	112.5 (13.6)	0.1
INR, mean (SD)	1.8 (0.1)	2.1 (0.2)	0.3
Albumin [g/dL], mean (SD)	3.1 (0.2)	2.5 (0.3)	0.1
**Complications of liver cirrhosis**			
Hepatic encephalopathy			
Grade 0-1, N (%)	18 (94.7)	11 (100)	0.7
Grade 2-3, N (%)	1 (5.3)	0 (0.0)	
Gastrointestinal bleeding			
N (%)	1 (5.3)	1 (9.1)	0.7
Infections			
N (%)	12 (63.2)	4 (36.4)	0.2
Ascites			
Minimal - moderate, N (%)	7 (36.8)	4 (36.4)	0.98
Massive, N (%)	12 (63.2)	7 (63.6)	
**Outcome**			
Mortality within 28 d, N (%)	1 (5.3)	1 (9.1)	0.7

P-values were calculated with One-way ANOVA, Kruskal-Wallis or Chi^2 -Test, as appropriate. AST, aspartate aminotransferase; CLIF-OF, chronic liver failure – organ failure; CRP, C-reactive protein; GGT, y-glutamyl transferase; Hb, hemoglobin; INR, international normalized ratio; MELD, model of end-stage liver disease.

SI Table 2. Baseline concentrations of patients with EASL-CLIF-ACLF versus mixed ACLF.

	**EASL-CLIF-ACLF (N=56)**	**Mixed-ACLF (N=43)**	**P-value**
Age [years], mean (SD)	57 (1.3)	51 (1.6)	**0.003**
Female gender; male gender, N (%)	15 (27); 41 (73)	16 (37); 27 (40)	0.3
Child Pugh Score, mean (SD)	8.7 (0.2)	10.6 (0.3)	**<0.0001**
CLIF OF score, mean (SD)	10.2 (0.3)	11.8 (0.3)	**0.0001**
MELD score, mean (SD)	24.1 (1.0)	34.8 (1.2)	**<0.0001**
**Etiology of liver cirrhosis**			
Viral, N (%)	5 (8.9)	1 (2.3)	0.2
NASH, N (%)	8 (14.3)	3 (6.7)	0.3
Alcoholic, N (%)	34 (60.7)	35 (81.4)	**0.03**
Cholestatic, N (%)	2 (3.6)	0 (0.0)	0.5
Others, N (%)	5 (8.9)	3 (7.0)	>0.9999
Mixed etiologies, N (%)	2 (3.6)	1 (2.3)	>0.9999
**Clinical biochemistry**			
Leukocytes [per nL], mean (SD)	8.6 (0.8)	13.9 (1.4)	**0.0003**
Hemoglobin [g/dL], mean (SD)	8.7 (0.2)	8.7 (0.3)	0.9
Platelets [per nL], mean (SD)	113.6 (10.6)	83.8 (7.0)	0.08
CRP [mg/dL], mean (SD)	4.8 (0.6)	4.6 (0.6)	0.5
Sodium [mmol/L], mean (SD)	134.8 (0.8)	131.9 (3.2)	0.7
Creatinine [mg/dL], mean (SD)	2.7 (0.2)	2.5 (0.2)	0.4
Bilirubin [mg/dL], mean (SD)	8.2 (1.5)	20.1 (1.7)	**<0.0001**
AST [U/L], mean (SD)	67.0 (7.4)	325.0 (172.6)	**0.0001**
GGT [U/L], mean (SD)	160.8 (24.4)	197.1 (49.3)	0.4
INR, mean (SD)	1.5 (0.06)	2.7 (0.2)	**<0.0001**
Albumin [g/dL], mean (SD)	3.1 (0.1)	3.3 (0.2)	0.4
**Complications of liver cirrhosis**			
Hepatic encephalopathy			
Grade 0, N (%)	38 (67.9)	8 (18.6)	**<0.0001**
Grade 1, N (%)	6 (10.7)	6 (14.0)	0.8
Grade 2, N (%)	9 (16.1)	8 (18.6)	0.8
Grade 3, N (%)	3 (5.4)	5 (11.6)	0.3
Gastrointestinal bleeding, N (%)	8 (14.3)	5 (11.6)	0.8
Infections, N (%)	35 (62.5)	28 (65.1)	0.8
Ascites			
No ascites, N (%)	12 (21.4)	5 (11.6)	0.3
Moderate, N (%)	9 (16.1)	12 (27.9)	0.2
Massive, N (%)	35 (62.5)	17 (60.5)	0.8
Mortality within 28 d, N (%)	6 (10.7)	17 (39.5)	**0.001**

P-values were calculated with One-way ANOVA, Kruskal-Wallis or Chi^2 -Test, as appropriate.
AST, aspartate aminotransferase; CLIF-OF, chronic liver failure – organ failure; CRP, C-reactive protein; GGT, y-glutamyl transferase; Hb, hemoglobin; INR, international normalized ratio; MELD, model of end-stage liver disease.
*57%, 37%, and 6% of patients with EASL-CLIF-ACLF had ACLF grade 1, 2, and 3, respectively.
[#]32%, 32%, and 36% of patients with mixed ACLF had ACLF grade 1, 2, and 3, respectively.

SI Table 3. Baseline concentrations of patients included in the OLINK-analyses.

	AD (N=85)	APASL-ACLF w/o prev. decomp (N=8)	EASL-CLIF-ACLF* (N=36)	Mixed-ACLF (N=28)#	P (AD vs APASL-ACLF)	P (AD vs EASL-CLIF-ACLF)	P (AD vs mixed-ACLF)	P (APASL-ACLF vs EASL-CLIF-ACLF)	P (APASL-ACLF vs mixed-ACLF)	P-value (EASL-CLIF-ACLF vs mixed-ACLF)
Age [years], mean (SD)	59 (11.2)	49 (11.2)	59 (9.7)	51 (10.4)	0.1	>0.99	**0.002**	0.2	>0.99	**0.01**
Female gender; male gender, N (%)	26 (31); 59 (69)	6 (75); 2 (25)	10 (28); 26 (72)	9 (32); 19 (68)	**0.02**	0.8	>0.99	**0.02**	**0.05**	0.8
Child Pugh Score, mean (SD)	8.3 (1.3)	10.0 (0)	8.7 (1.3)	10.3 (1.4)	>0.99	>0.99	**0.0003**	>0.99	>0.99	**0.03**
CLIF OF score, mean (SD)	7.9 (1.4)	10.3 (1.8)	10.1 (1.6)	12.1 (2.4)	**0.003**	**<0.0001**	**<0.0001**	>0.99	>0.99	0.1
MELD score, mean (SD)	14.5 (4.2)	24.1 (2.4)	24.1 (7.6)	36.5 (7.9)	**0.001**	**<0.0001**	**<0.0001**	>0.99	0.8	0.0098
**Etiology of liver cirrhosis**										
Viral, N (%)	8 (9)	0 (0)	3 (8)	1 (4)	>0.99	>0.99	0.4	>0.99	>0.99	0.6
NASH, N (%)	8 (9)	0 (0)	7 (19)	0 (0)	>0.99	0.1	0.2	0.3	>0.99	**0.02**
Alcoholic, N (%)	40 (47)	7 (87.5)	18 (50)	24 (86)	0.06	0.8	**0.0003**	0.1	>0.99	**0.004**
Cholestatic, N (%)	3 (4)	0 (0)	1 (3)	0 (0)	>0.99	>0.99	0.6	>0.99	>0.99	>0.99
Others, N (%)	18 (21)	0 (0)	4 (11)	2 (7)	0.3	0.3	0.2	>0.99	>0.99	0.7
Mixed etiologies, N (%)	8 (9)	1 (12.5)	3 (8)	1 (4)	0.6	>0.99	0.4	0.6	0.4	0.6
**Clinical biochemistry**										
Leukocytes [per nL], mean (SD)	7.5 (4.7)	12.7 (8)	9.4 (6.3)	13.7 (7.3)	0.3	>0.99	**<0.0001**	>0.99	>0.99	**0.03**
Hemoglobin [g/dL], mean (SD)	9.9 (2.5)	9.7 (1.4)	8.9 (1.8)	9.3 (1.8)	>0.99	0.099	>0.99	>0.99	>0.99	>0.99
Platelets [per nL], mean (SD)	138.6 (90.1)	137 (85.3)	123.6 (80.5)	96.4 (47)	>0.99	>0.99	0.2	>0.99	>0.99	>0.99
CRP [mg/dL], mean (SD)	3.0 (3.2)	3.0 (2.6)	5.6 (5)	4.5 (3.6)	>0.99	**0.01**	0.06	>0.99	>0.99	>0.99
Creatinine [mg/dL], mean (SD)	1.2 (0.4)	1.0 (0.3)	2.7 (1.1)	2.9 (1.4)	0.8	**<0.0001**	**<0.0001**	**<0.0001**	**<0.0001**	0.99
Bilirubin [mg/dL], mean (SD)	3.1 (3.6)	16.7 (11.6)	8.9 (12.3)	21.8 (12.2)	**0.0007**	0.8	**<0.0001**	**0.02**	>0.99	**<0.0001**
AST [U/L], mean (SD)	87.9 (98.2)	81.3 (62.6)	63 (49.7)	185.6 (157.4)	>0.99	>0.99	**0.004**	>0.99	0.5	**0.0009**
GGT [U/L], mean (SD)	283.7 (589.8)	243 (252.5)	185.8 (207.4)	268.1 (381.8)	>0.99	>0.99	>0.99	>0.99	>0.99	>0.99
INR, mean (SD)	1.4 (0.3)	1.9 (0.2)	1.6 (0.5)	2.8 (1.7)	**0.001**	**0.04**	**<0.0001**	0.2	>0.99	**<0.0001**
Albumin [g/dL], mean (SD)	3.0 (0.6)	3.1 (0.4)	3.0 (0.6)	3.0 (0.8)	0.98	0.96	>0.99	0.9	0.98	0.99
Hepatic encephalopathy										
Grade 0-1, N (%)	75 (88)	8 (100)	27 (83)	16 (57)	0.6	0.4	**0.0008**	0.3	**0.03**	**0.05**
Grade 2-3, N (%)	10 (12)	0 (0)	6 (18)	12 (43)						
Gastrointestinal bleeding, N (%)	13 (16)	0 (0)	4 (11)	3 (11)	0.6	0.8	0.8	>0.99	>0.99	>0.99
Infections, N (%)	38 (45)	6 (75)	24 (69)	19 (68)	0.06	**0.004**	**0.008**	>0.99	>0.99	>0.99
Ascites										
Mild-Moderate, N (%)	43 (51)	2 (25)	12 (35)	10 (37)	0.3	0.2	0.3	0.7	0.7	>0.99
Massive, N (%)	42 (49)	6 (75)	22 (65)	17 (61)						
**Outcome**										
Mortality within 28 d, N (%)	2 (2)	0 (0)	4 (11)	11 (39)	>0.99	0.06	**<0.0001**	>0.99	0.08	**0.02**

P-values were calculated with One-way ANOVA, Kruskal-Wallis or Chi^2 -Test, as appropriate.
AST, aspartate aminotransferase; CLIF-OF, chronic liver failure – organ failure; CRP, C-reactive protein; GGT, y-glutamyl transferase; Hb, hemoglobin; INR, international normalized ratio; MELD, model of end-stage liver disease.
*52%, 43%, and 5% of patients with EASL-CLIF-ACLF had ACLF grade 1, 2, and 3, respectively.
#38,5%, 38,5%, and 20% of patients with mixed ACLF had ACLF grade 1, 2, and 3, respectively.

SI Table 4. Baseline concentrations of patients included in the analyses of immune cell phenotypes.

	AD (N=6)	APASL-ACLF w/o prev. decomp (N=7)	EASL-CLIF-ACLF (N=6)*	P (AD vs APASL-ACLF)	P (AD vs EASL-CLIF-ACLF)	P (APASL-ACLF vs EASL-CLIF-ACLF)
Age [years], mean (SD)	53 (8.4)	53 (7.6)	53 (5.7)	0.97	0.99	0.99
Female gender; male gender, N (%)	0 (0%), 6 (100)	1 (14.3), 6 (85.7)	0 (0%), 6 (100)	>0.99	>0.99	>0.99
Child Pugh Score, mean (SD)	8.3 (1.4)	10.4 (1.6)	9.2 (1.5)	0.06	0.6	0.3
CLIF OF score, mean (SD)	7.3 (1.2)	7.7 (0.8)	9.3 (1.8)	0.85	**0.04**	0.09
MELD score, mean (SD)	16.9 (6.2)	24.3 (3.6)	30.5 (5.6)	**0.048**	**0.001**	0.1
**Etiology of liver cirrhosis**						
Viral, N (%)	1 (17)	0 (0)	0 (0)	0.5	0.5	>0.99
NASH, N (%)	2 (33)	0 (0)	1 (17)	0.2	>0.99	0.5
Alcoholic, N (%)	2 (33)	4 (57)	5 (83)	0.6	0.2	0.6
Cholestatic, N (%)	0 (0)	0 (0)	0 (0)	-	-	-
Others, N (%)	0 (0)	2 (29)	0 (0)	0.5	>0.99	0.5
Mixed etiologies, N (%)	1 (17)	1 (14)	0 (0)	>0.99	>0.99	>0.99
**Clinical biochemistry**						
Leukocytes [per nL], mean (SD)	6.8 (3.5)	7.7 (2.3)	6.7 (3.3)	>0.99	>0.99	>0.99
Hemoglobin [g/dL], mean (SD)	11.1 (2.6)	10 (2.5)	9.2 (1.9)	0.7	0.4	0.8
Platelets [per nL], mean (SD)	162.7 (203.7)	86 (37.5)	73.5 (57.4)	>0.99	>0.99	>0.99
CRP [mg/dL], mean (SD)	2.3 (4)	2.1 (1.5)	2.6 (1.7)	0.6	0.4	>0.99
Creatinine [mg/dL], mean (SD)	1 (0.3)	1.1 (0.3)	5.6 (1.7)	>0.99	**0.009**	**0.008**
Bilirubin [mg/dL], mean (SD)	4.2 (3.1)	15.9 (10.1)	11.7 (14.5)	**0.04**	>0.99	0.2
AST [U/L], mean (SD)	52.8 (20.1)	148.3 (134.8)	104 (91.4)	0.07	0.8	0.8
GGT [U/L], mean (SD)	193.3 (194.8)	80.9 (44.4)	123.7 (107.6)	0.3	0.6	0.8
INR, mean (SD)	1.6 (0.5)	1.9 (0.3)	1.8 (0.2)	0.5	0.7	0.9
Albumin [g/dL], mean (SD)	3.3 (0.4)	2.9 (0.9)	3.4 (1)	0.8	0.9	0.6
Hepatic encephalopathy						
Grade 0-1, N (%)	6 (100)	6 (86)	4 (67)	>0.99	0.5	0.6
Grade 2-3, N (%)	0 (0)	1 (14)	2 (33)			
Gastrointestinal bleeding, N (%)	0 (0)	1 (14)	0 (0)	>0.99	>0.99	>0.99
Infections, N (%)	0 (0)	4 (57)	3 (50)	0.07	0.2	>0.99
Ascites						
Mild-Moderate, N (%)	4 (67)	4 (57)	2 (33)	>0.99	0.6	0.6
Massive, N (%)	2 (33)	3 (43)	4 (67)			
**Outcome**						
Mortality within 28 d, N (%)	0 (0)	1 (14.3)	0 (0)	>0.99	>0.99	>0.99

P-values were calculated with One-way ANOVA, Kruskal-Wallis or Chi^2 -Test, as appropriate.
AST, aspartate aminotransferase; CLIF-OF, chronic liver failure – organ failure; CRP, C-reactive protein; GGT, y-glutamyl transferase; Hb, hemoglobin; INR, international normalized ratio; MELD, model of end-stage liver disease.
*33%, 33%, and 33% of patients with EASL-CLIF-ACLF had ACLF grade 1, 2, and 3, respectively.

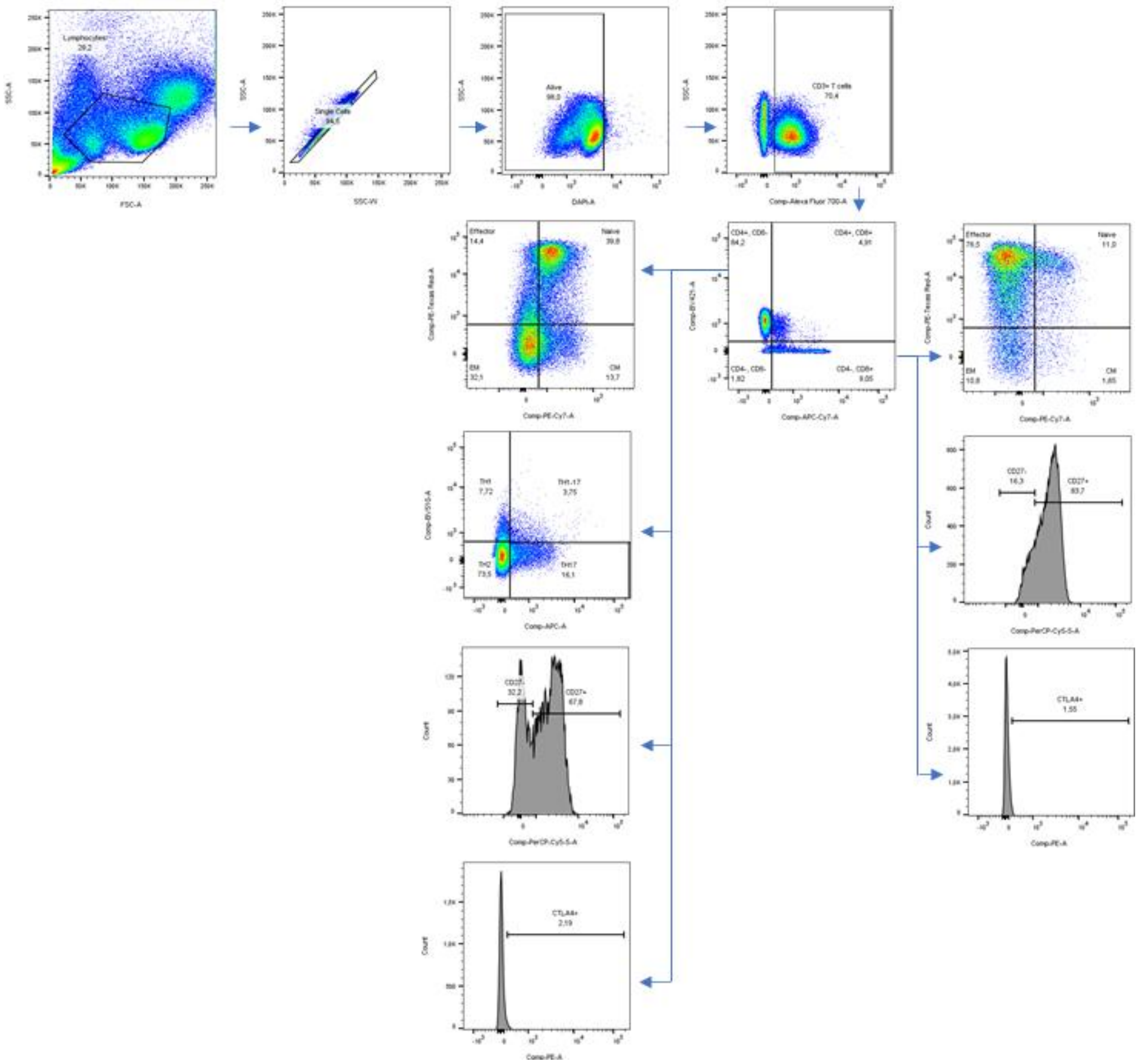

SI Figure 1. Gating strategy for T-lymphocytes. First, in a SSC-A / FSC-A scatter the lymphocyte population is gated, followed by singlet gating (SSC-A / SSC-W). After that, dying or dead cells are excluded according to Zombie UV expression. Alive T-lymphocytes are identified through CD3-expression and then subdivided into CD4+ and CD8+ T-lymphocytes. Effector and naïve subsets are identified according to their CCR7 and CD45RA expression (where naïve cells are CCR7+CD45RA+, central memory cells are CCR7+CD45RA-, effector memory cells are CCR7-CD45RA- and effector cells are CCR7-CD45RA+). T-helper subsets can also be subdivided into Th1 cells (CXCR3+CCR6-), Th1/Th17 cells (CXCR3+CCR6+), Th17 cells (CXCR3-CCR6+), and other Th cells (CXCR3-CCR6-). All additional activation and inhibitory markers are gated on CD4+ and CD8+ T-cells.

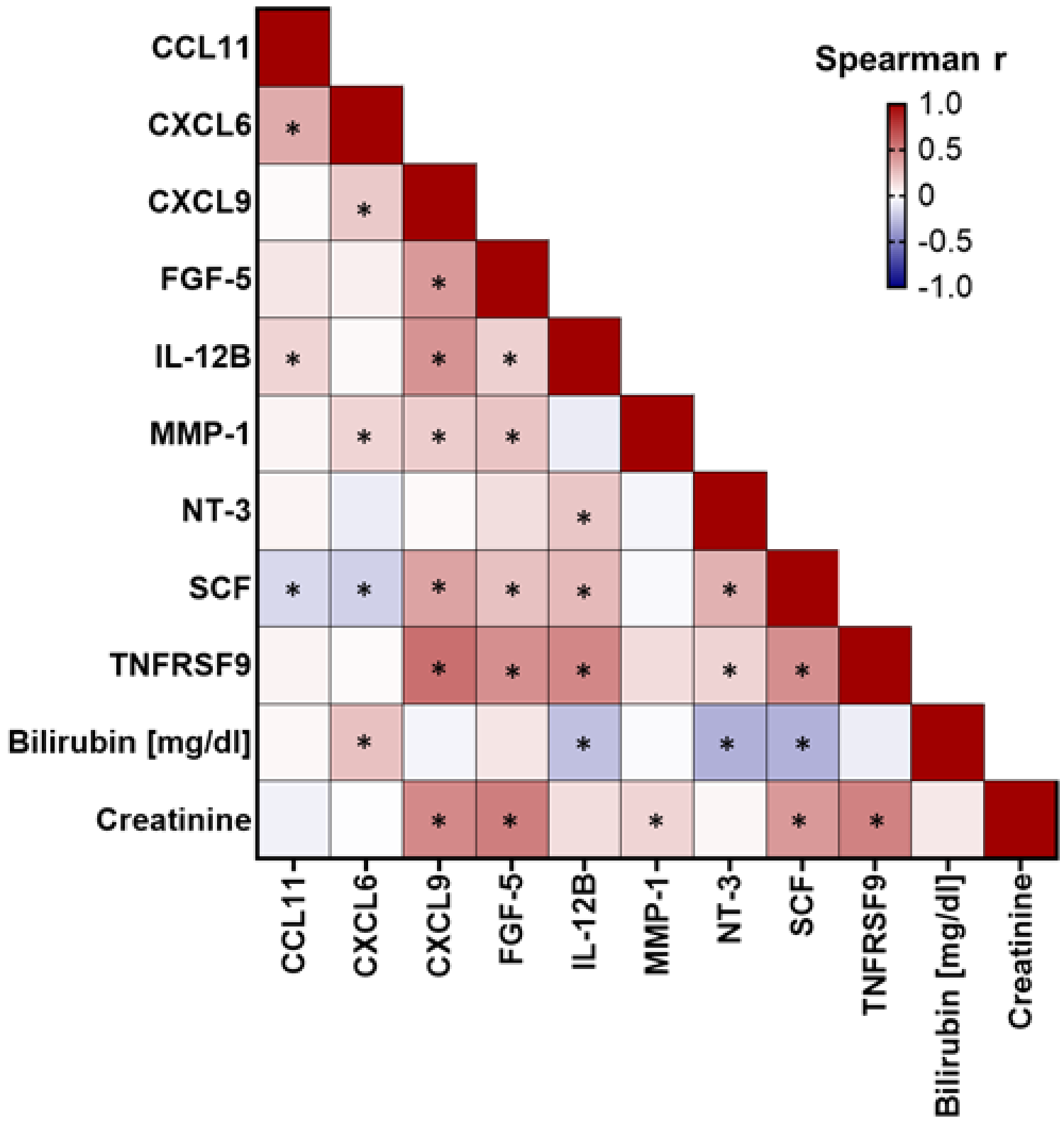

SI Figure 2. Correlations of inflammatory mediators with bilirubin and serum creatinine.

*P ≤ 0.05, **P ≤ 0.01.

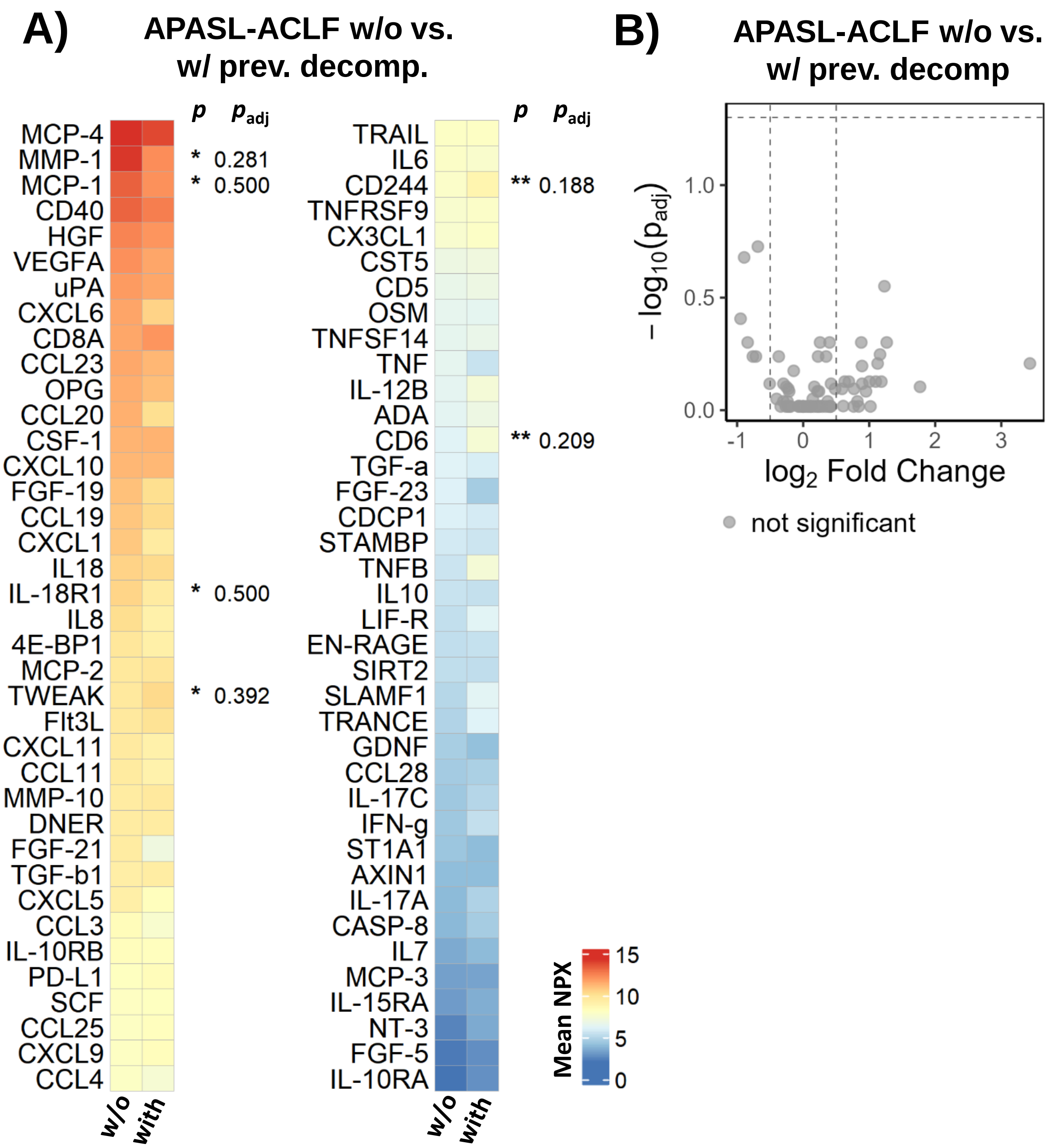

SI Figure 3. Patterns of systemic inflammation according to "APASL-ACLF" without versus with previous decompensation. Heat-maps (A) and volcano blots (B) of 76 plasma proteins and inflammatory mediators which were quantified by proximity extension analysis assay (Olink), in patients with APASL-ACLF with versus without prior decompensation. Unadjusted P values are shown in the left column (*P ≤ 0.05, **P ≤ 0.01), while P values after Benjamini-Hochberg FDR correction are shown in the right column.

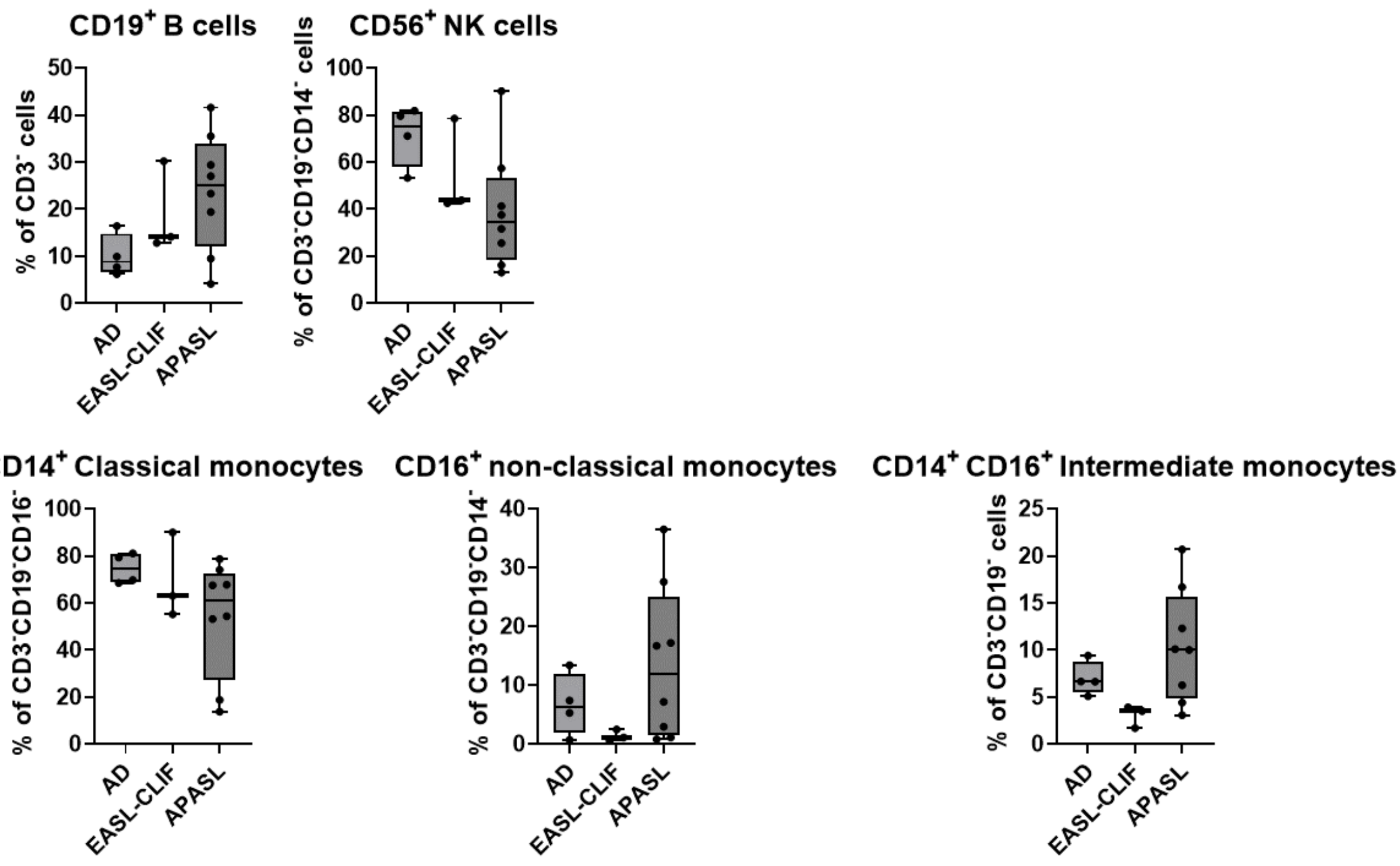

SI Figure 4. Frequencies of B cells, NK cells, and monocytes according to the type of AD / ACLF.

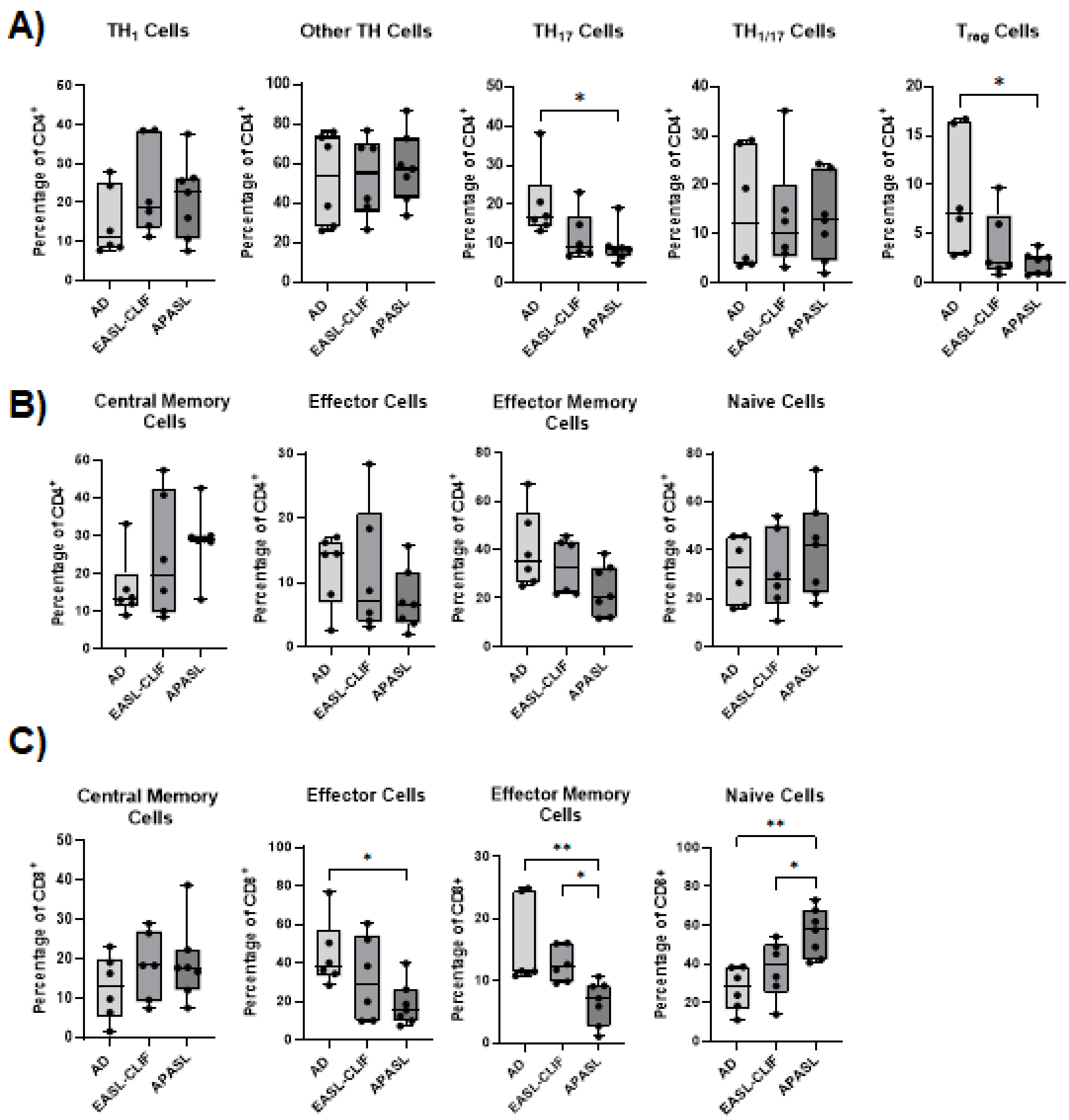

SI Figure 5. Frequencies of CD4+ and CD8+ T cell subpopulations according to the type of AD / ACLF. A) Quantification of Th-cell subsets (Th1, Th17, Th1/17, other Th cells) and differentiation of Treg cells (CD25+CD127dim). B) Identification of central memory cells, effector cells, effector memory cells and naïve cells of CD4+ T-Cells. C) Identification of central memory cells, effector cells, effector memory cells and naïve cells of CD8+ T-cells.
